# Contact-free experimental determination of the static flexural spring constant of cantilever sensors using a microfluidic force tool

**DOI:** 10.3762/bjnano.7.43

**Published:** 2016-03-30

**Authors:** John D Parkin, Georg Hähner

**Affiliations:** 1 EaStCHEM School of Chemistry, University of St. Andrews, North Haugh, St. Andrews, KY16 9ST, UK

**Keywords:** AFM, cantilever sensors, microfluidic force tool, spring constant

## Abstract

Micro- and nanocantilevers are employed in atomic force microscopy (AFM) and in micro- and nanoelectromechanical systems (MEMS and NEMS) as sensing elements. They enable nanomechanical measurements, are essential for the characterization of nanomaterials, and form an integral part of many nanoscale devices. Despite the fact that numerous methods described in the literature can be applied to determine the static flexural spring constant of micro- and nanocantilever sensors, experimental techniques that do not require contact between the sensor and a surface at some point during the calibration process are still the exception rather than the rule. We describe a noncontact method using a microfluidic force tool that produces accurate forces and demonstrate that this, in combination with a thermal noise spectrum, can provide the static flexural spring constant for cantilever sensors of different geometric shapes over a wide range of spring constant values (≈0.8–160 N/m).

## Introduction

Micro- and nanocantilevers are routinely employed as probes down to the nanometer scale. In atomic force microscopy (AFM), microcantilever sensors are used, for example, to image the topography of surfaces and to map mechanical properties with nanometer resolution [1–3]. In addition, so-called force curves can reveal information about the interaction between the AFM tip and the surface, thus providing information about local interactions [4]. Cantilever structures also form an integral part of micro- and nanoelectromechanical systems (MEMS and NEMS) [5–7] and can be employed as freestanding sensors [8–13].

In many applications where a cantilever-type sensor is involved, the calibration of the sensor stiffness (spring constant, *k*) is a prerequisite for obtaining quantitative data. Several methods describing how the static flexural spring constant can be calibrated have been reported in the literature, in particular in relation to AFM [14–15]. However, many of the experimental approaches have drawbacks or limitations and cannot easily be extended to an array of cantilevers. One of the major drawbacks of many of the available experimental techniques is the requisite contact between the probe and surface during the calibration process, which can damage the AFM tip. Therefore, methods that do not involve any contact are highly desirable.

For softer cantilevers (*k* < 5 N/m) and simple geometries, such as rectangular-shaped beams, several calibration methods have been demonstrated to work well [16–18]. However, for stiff cantilevers (*k* > 20 N/m) and “unusual” geometric shapes, the determination of the spring constant is still a challenge [14,19]. The most direct method for the determination of the static spring constant comprises the application of a well-defined force to the sensor and measuring the resulting deflection. One way to do this is by pressing the cantilever against a balance [20–21] or a precalibrated cantilever [22–23]. However, the disadvantage is that the tip is in mechanical contact with a hard surface and can therefore be damaged [24–25]. Furthermore, these methods are appropriate for cantilevers with a spring constant typically greater than ≈1 N/m [20] and less than ≈10 N/m [26]. The application of forces other than mechanical force, such as magnetic or electrostatic force, requires modification of the cantilever, for example, with a magnetic coating. This can pose a problem in itself because of the small dimensions of the cantilever structure. In addition, the coating can cause stress, resulting in a static deflection of the sensor beam. It is therefore desirable to have a universal force tool that can exert well-defined forces on all types of cantilever sensors independent from their physical and chemical properties. A microfluidic flow tool has been previously employed in connection with cantilever spring constant determination [27–30], and it was shown that forces due to the flow from a microfluidic channel can be exploited to determine the dynamic flexural spring constants [29] as well as the torsional and lateral spring constants [30]. In the following, we describe a method to determine the static flexural spring constant for cantilevers of any geometric shape. The approach can be applied to very soft as well as very stiff cantilevers. We demonstrate that a microfluidic flow can provide accurate forces and allows the static spring constant of cantilever sensors to be determined with high precision and without any contact between the sensor and a surface. We show the applicability of the method for spring constants in the range of 0.8 N/m to ≈160 N/m.

## Method

### Determination of the static flexural spring constant

The static flexural spring constant depends on the force distribution, 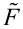, applied to the cantilever sensor as well as the position, *x*, along the beam where the resulting deflection, 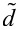
*,* is measured [31–32]. The corresponding general expression for the static spring constant, 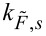, is:

[1]
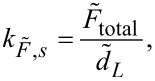


where 
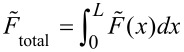
 is the total force exerted by the force distribution, 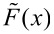, over the cantilever length, *L*, and 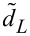 is the resulting static deflection of the cantilever measured at position *L*.

In AFM, a point load 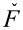 applied at the position of the probe tip results in a deflection 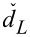 of the cantilever that is typically measured at or close to the free end of the beam. The static spring constant resulting from this point load, 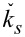, is therefore given by

[2]
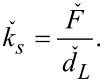


The spring constant for any force distribution, 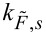, can be converted to an equivalent spring constant for a point load, 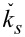, applied at the position of the probe tip by a generalization of the procedure described in [31]. In general, there is no analytical expression for the conversion factor 
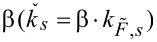
, but it can be determined numerically. Experimentally measuring the spring constant 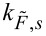 therefore allows 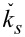 to be determined if the conversion factor β is known.

The static spring constant for a point load, 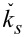, can be obtained from the dynamic spring constant of the first flexural mode 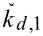 if the dynamic-to-static spring constant conversion factor γ is known [32–33]: 
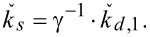
 The dynamic spring constant is related to the mean-squared displacement, 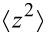, of the fundamental flexural mode of the cantilever: 
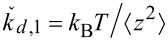
 [16], where *k*_B_ is the Boltzmann constant, and *T* is the absolute temperature. The mean-squared displacement 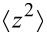 is obtained from the area under the thermal resonance curve. Combining these equations gives

[3]
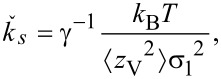


where *z*_V_ is the photodiode signal in native units of the instrument (volts) and σ_1_ is the optical lever sensitivity for thermal oscillations [34–35]. Knowledge of σ_1_ is required for calibration of the spring constant via the thermal noise method [16]. It can be experimentally obtained, for example, from a force curve.

Similarly, the measured spring constant for a force distribution 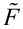 is

[4]
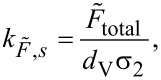


where *d*_V_ is the deflection in the native units of the instrument (volts) and σ_2_ is the optical lever sensitivity for bending under the force distribution 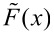. Note that even without knowledge of σ_1_ and σ_2_, the dynamic-to-static sensitivity ratio α = σ_1_/σ_2_ can still be theoretically obtained. This conversion is similar to the dynamic-to-static optical lever sensitivity conversion required in the thermal noise method [33].

From the photodiode signal, the deflection for a total force and the peak area under a thermal noise curve are obtained in units of volts and volts squared, respectively. The spring constant 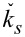 can then be determined without knowledge of the individual optical lever sensitivities σ_1_ and σ_2_ by combining Equation 3 and Equation 4 and rearranging them as

[5]
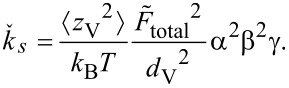


## Experimental

### Setup and measurements

Experiments were performed with a commercial Bruker Dimension FastScan AFM system (Bruker, Santa Barbara, CA, USA). In our setup, a custom-built, smooth parallel plate microchannel of height ≈100 μm and length 4.5 mm was used [27,29–30]. An accurate value of the channel height was obtained by contacting the free end of a cantilever on the bottom surface of the channel with the channel aligned parallel to the cantilever length and measuring the distance to the top of the channel by lifting the cantilever with the AFM microstepper motor until it contacted the top surface of the channel. This gave a value of 106 μm.

**Figure 1:**
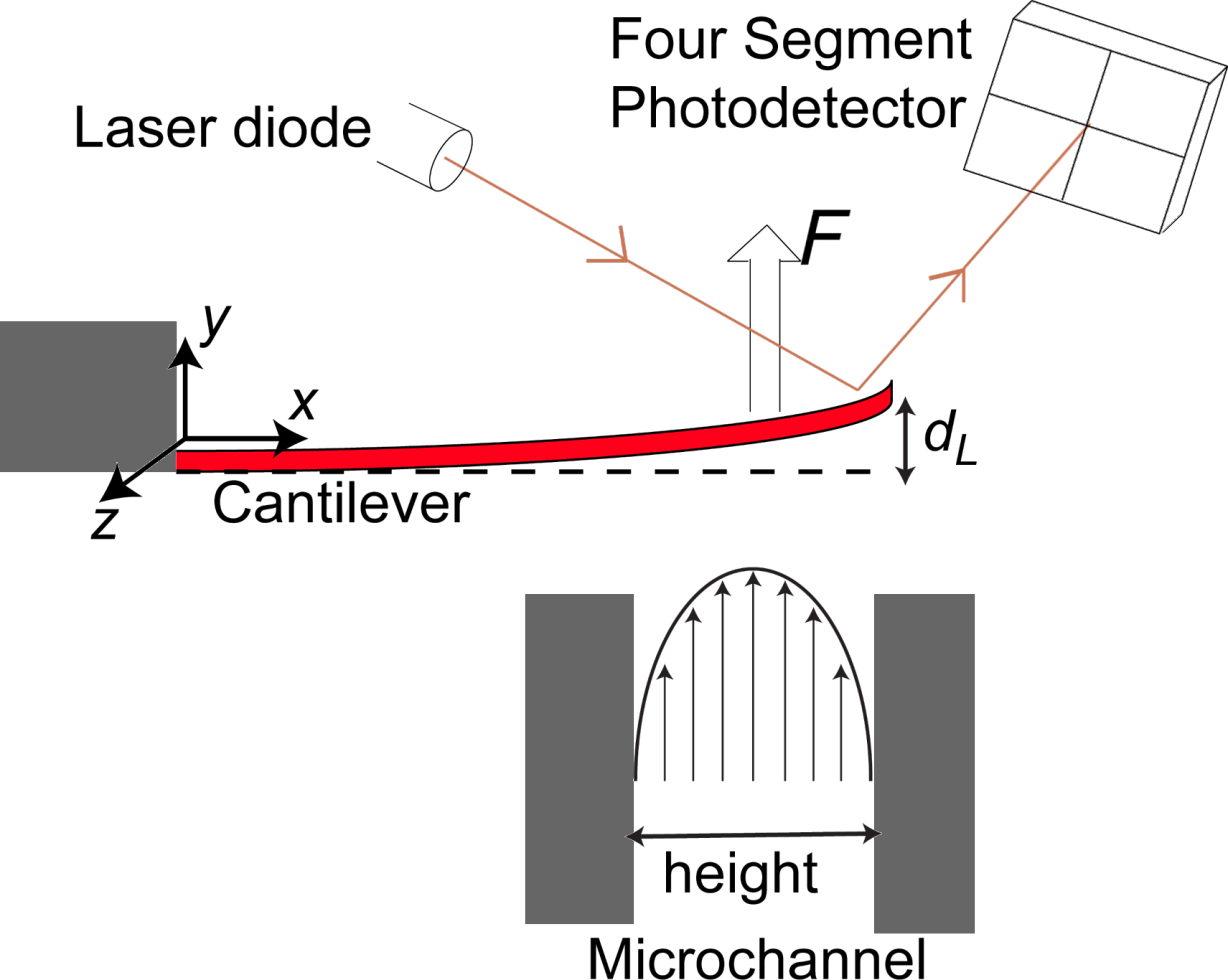
Schematic side view of the microchannel with Poiseuille profile of the fluid and a cantilever that bends due to forces exerted by the fluid.

For the measurements, the channel was fixed on the sample stage of the AFM and positioned such that fluid flow from its exit interacted with the cantilever as illustrated in Figure 1. The channel was aligned such that the free end of the cantilever was level with the edge of the channel and 100 μm above the channel exit. This alignment was chosen because of the ease of reproducibility.

Nitrogen gas was used as the working fluid. Pressure differences were applied to the microchannel to drive the flow, establishing stable Poiseuille velocity profiles [27]. The maximum pressure applied to the channel depended on the cantilever studied. The highest pressure used was ≈3.5 kPa, resulting in a nitrogen velocity value of about ≈62 m/s in the channel mid-line [28], corresponding to a laminar, incompressible flow [36].

The forces applied to the cantilever by the fluid flow cause a static flexural bending [30]. The bending of the cantilever as a function of fluid velocity was recorded by reading out the photodiode signal of the AFM with a self-coded LabVIEW routine via a signal access module (SAM-V, Bruker, CA, USA) and an external interface (USB-6251, National Instruments). The power spectral density of the thermal noise was obtained with the Bruker software. The peak area, resonant frequency and *Q*-factor of the thermal noise spectra were determined with a self-coded MATLAB routine by fitting Lorentzian curves to the resonance peaks of the first flexural modes. Force curves, to calibrate the deflection sensitivity (σ_1_) for the thermal noise method, were recorded on clean sapphire substrates.

### Cantilevers studied

**Figure 2:**
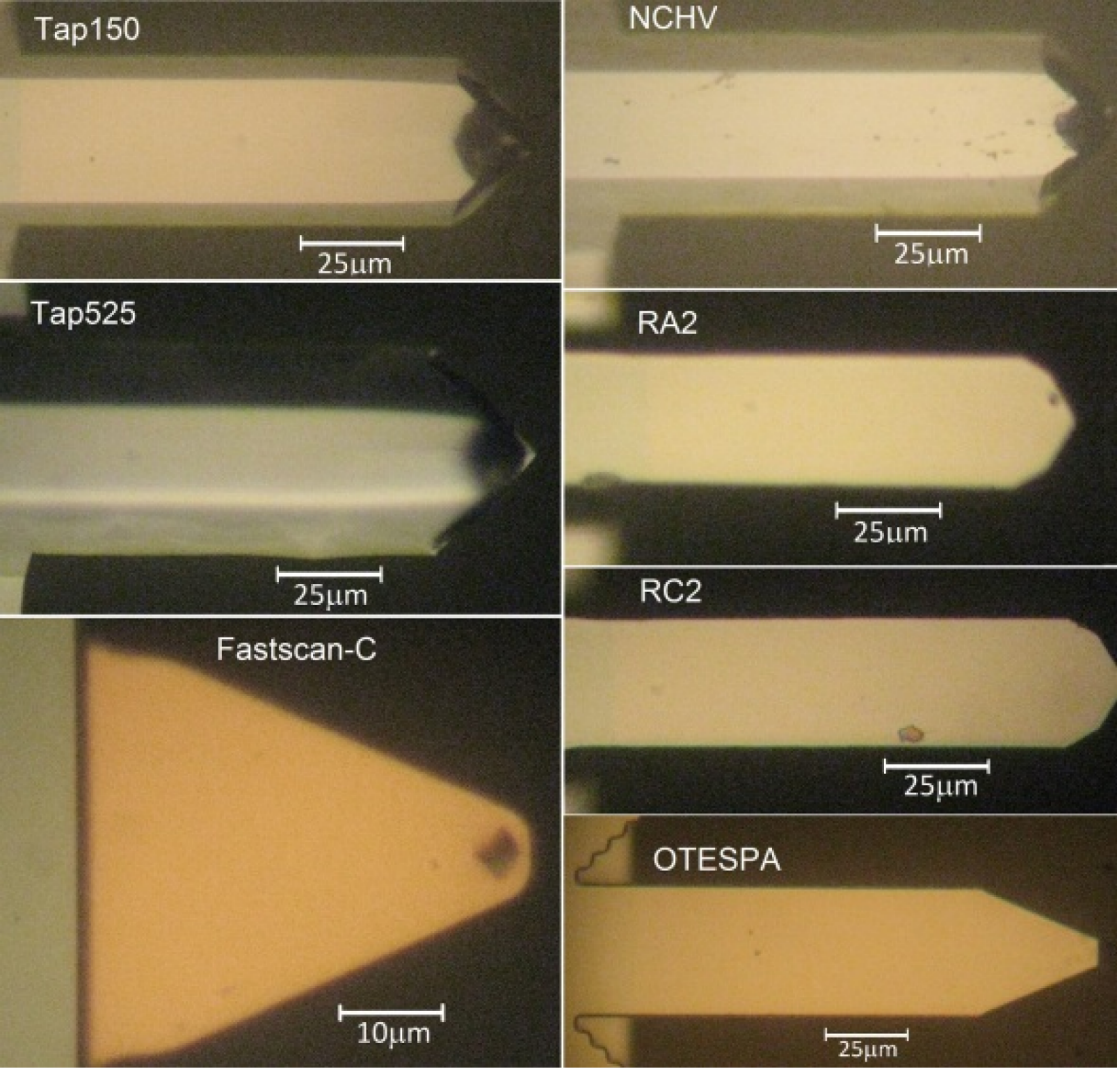
Optical images of the AFM cantilevers studied showing their plan view geometries (view from the tip side). Details of the cantilever dimensions are given in the Results section.

To test our approach, a range of commercially available cantilevers were studied (see Figure 2). RA2 and RC2 are tipless cantilevers (Mikromasch, Tallinn, Estonia) while OTESPA, Tap150, NCHV, Tap525, and Fastscan-C all have tips attached (Bruker, Santa Barbara, USA). Some cantilevers had metal coatings to increase the reflectivity of the laser: Tap150, Tap525 and OTESPA were aluminum-coated and Fastscan-C was gold-coated. RA2, RC2 and NCHV had no metal coating.

## Results

### Cantilever dimensions, resonant frequency and *Q*-factor

Table 1 summarizes the geometrical dimensions, resonant frequency and *Q*-factor of the cantilevers studied. Here, “Nominal” refers to the information provided by the manufacturers. The actual plan view dimensions of all microcantilevers were determined with an Olympus optical microscope. Some of the cantilevers had a trapezoidal cross-section (see Figure 2), in which case both the width at the top and the bottom were determined.

**Table 1:** Nominal and experimentally determined geometric dimensions (width, *w*, thickness, *t*, full length, *L*, and length of picketed end, *l*, all given in μm), fundamental frequency, *f* (kHz), and *Q*-factor of the cantilevers studied.

	RA2	RC2	OTESPA	Tap150	NCHV	Tap525	Fastscan-C

*w*_nominal_	35	35	40	30	40	40	40 (footprint)
*w*_exp,top_	31.0	31.0	42.5	40.0	42.0	52.0	42.0 (fixed end) 6.0 (free end)
*w*_exp,bottom_	31.0	31.0	41.0	30.0	25.5	24.0	42.0 (fixed end) 6.0 (free end)
*t*_nominal_	2	2	3.7	1.85	4	6.25	0.3
*L*_nominal_	110	130	160	125	125	125	40
*L*_exp_	110.0	124.5	149.0	124.0	121.0	123.0	44.5
*l*_exp_	15.0	15.0	40.0	15.0	19.0	26.0	–
*f*_1,nominal_	210	150	300	150	320	525	300
*f*_1,exp_	231.658	162.283	297.540	173.952	324.167	512.316	210.916
*Q*-factor	268	222	470	262	439	773	102

### Determination of the force distribution and the conversion factors α, β, and γ

**Figure 3:**
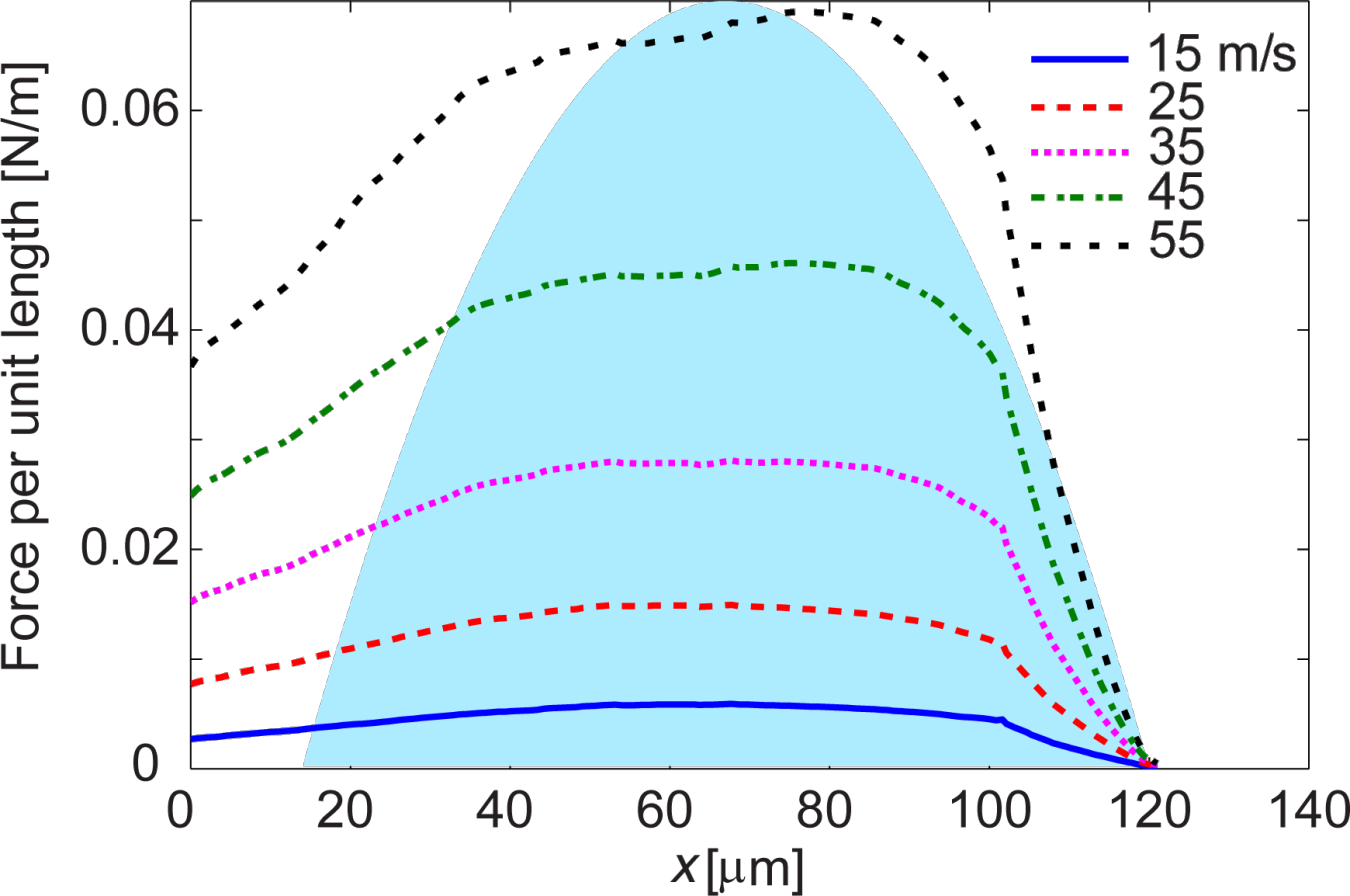
Force per unit length as a function of the fluid speed experienced by cantilever NCHV. The fixed end of the cantilever is at *x* = 0. The shaded area is a sketch of the fluid speed profile escaping the channel and also indicates the boundaries of the microchannel exit along the *x*-axis.

To provide information about the interaction between the fluid flow escaping from the microchannel and the cantilevers, we performed finite element method simulations with COMSOL Multiphysics [37]. The mesh independence of the results was confirmed by mesh refinement. The forces applied to the cantilever by the fluid flow were extracted from the simulations. Figure 3 displays the force per unit length for different fluid speeds at the center of the microchannel applied to a cantilever using the example of NCHV (see Table 1).

The total force 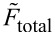 experienced by the cantilever sensor was obtained by integrating the force distribution over the cantilever length. The conversion factor α was determined with a self-coded MATLAB routine. This conversion factor is similar in nature to a factor that is also required for calibration with the thermal noise method [33].

For the conversion factor β, the static deflection of the cantilever beam as a function of the force profile for different fluid speeds and for the force profiles obtained from the simulations was determined with a self-coded MATLAB routine following the procedures described in [31–32]. In addition, the bent shape was also determined for a point load applied at the free end of the cantilever.

**Figure 4:**
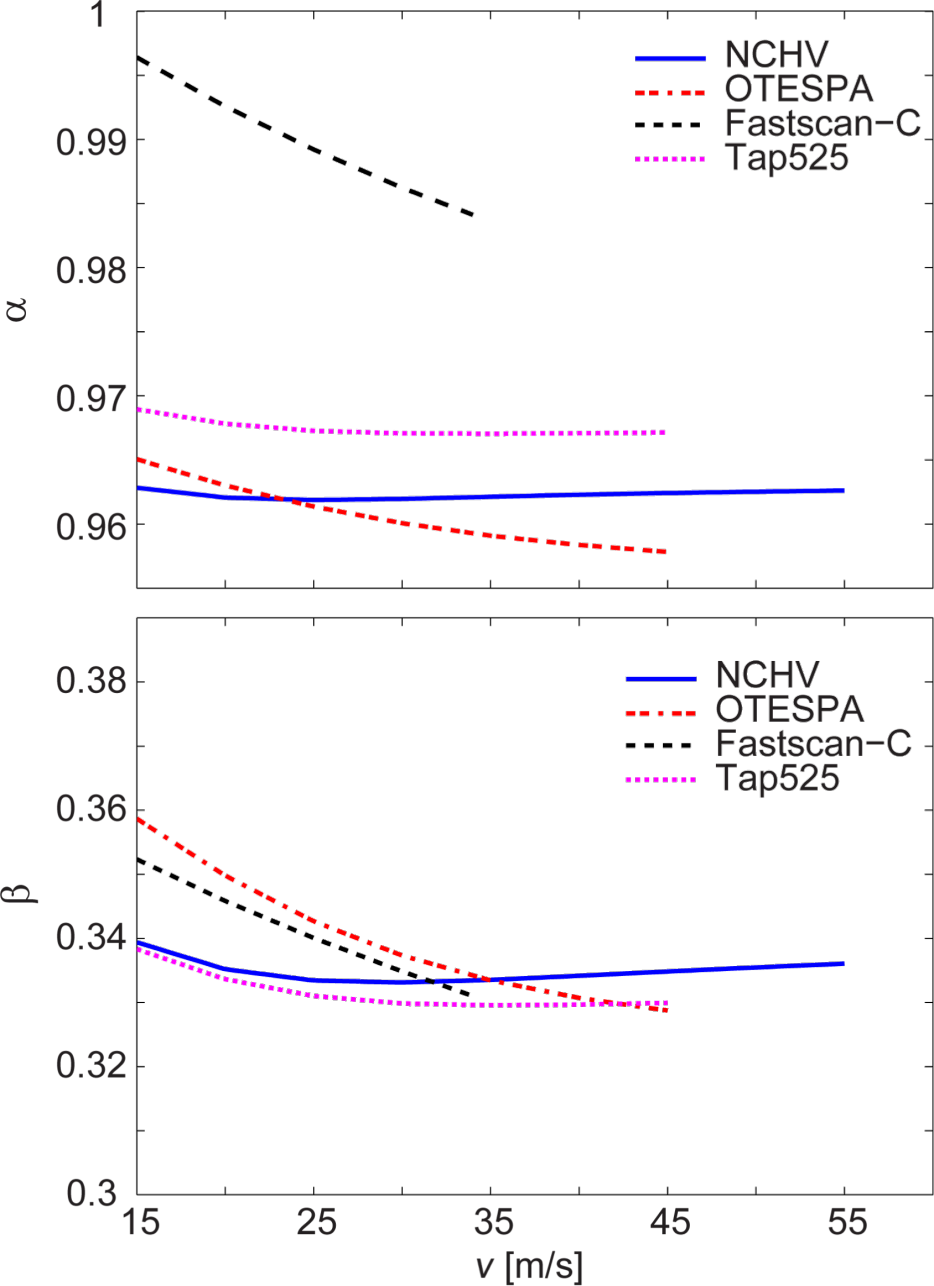
Fluid-flow-dependent conversion factors α and β for some of the cantilevers studied. The conversion factors of cantilevers RA2, RC2 and Tap150 (not shown) showed a behavior very similar to that of NCHV and Tap525.

The conversion factors α and β depend on the force distribution. They are therefore dependent on the fluid speed if the force distribution changes with speed. Figure 4 displays α and β for some of the cantilevers studied and for fluid speeds above 15 m/s. The conversion factors are fairly constant for all cantilevers and for the alignment chosen in our experiment. The behavior of the conversion factors for the cantilevers not shown (RA2, RC2, Tap150) was very similar to that shown for NCHV and Tap525.

In Table 2, the mean values of α and β together with their standard deviations for the speed range above 15 m/s are reported. The standard deviation of α is well below 1% over this range for all cantilevers, while it is typically less than 1% for β with the exception of FastScan-C (2.1%) and OTESPA (2.3%), which are the most picketed cantilevers studied. The conversion factor γ, also given in Table 2, is identical to the one required in the thermal noise calibration method. We determined γ for the different geometries of the cantilevers with self-coded MATLAB routines. For many cantilever geometries, this can also be found in the literature (see for example [33]). The geometric data reported in Table 1 was used for the calculations of all conversion factors.

**Table 2:** Conversion factors with their standard deviations (Δ), and nominal (manufacturer quoted) and experimentally determined spring constant values. No individual error estimates are stated for the thermal noise measurements but are typically found to be in the range of 10–20% [14,34].

	RA2	RC2	OTESPA	Tap150	NCHV	Tap525	Fastscan-C

χ	1.1290	1.1236	1.1916	1.1237	1.1378	1.1620	1.1801
α Δα	0.9773 0.0002	0.9762 0.0002	0.9606 0.0021	0.9630 0.0004	0.9623 0.0003	0.9674 0.0005	0.9355 0.0074
β Δβ	0.3541 0.0012	0.3811 0.0013	0.3385 0.0078	0.3481 0.0024	0.3342 0.0011	0.3310 0.0019	0.3423 0.0072
γ	1.0490	1.0470	1.0740	1.0470	1.0530	1.0630	1.0900
*k*_nom_ (*k*_min_; *k*_max_)	7.5 (3.5;12.5)	4.5 (2.5; 8.5)	26 (8.4; 57)	5 (2.5; 10)	42 (20; 80)	200 (100; 400)	0.8 (0.4; 1.2)
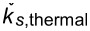	7.7	5.26	26.5	10.3	36.7	123.8	0.63
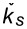	8.5 ± 0.3	4.35 ± 0.09	33.1 ± 2.4	8.3 ± 0.3	42.4 ± 2.1	154.2 ± 2.4	0.88 ± 0.07

### Deflection under fluid flow and determination of the static flexural spring constant

**Figure 5:**
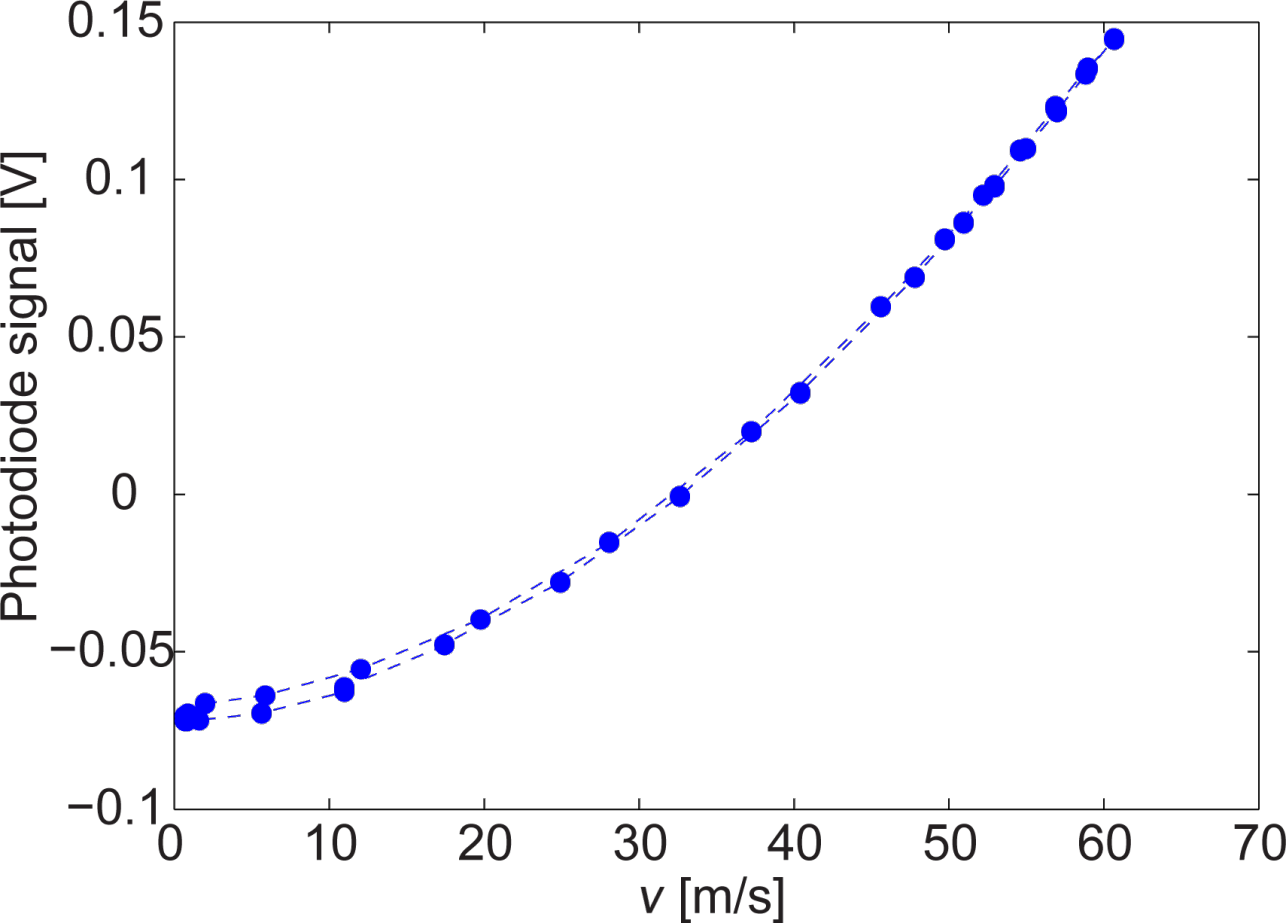
Cantilever deflection of NCHV under fluid flow from the microchannel measured at the free end of the cantilever.

Figure 5 shows a typical deflection curve for cantilever NCHV as a function of the fluid flow speed. The pressure applied to the channel, and hence the fluid flow speed, was first increased and then decreased in the experiment. A slight hysteresis can be observed in the deflection curve for fluid speeds below ≈15 m/s, corresponding to pressure values of <0.8 kPa.

**Figure 6:**
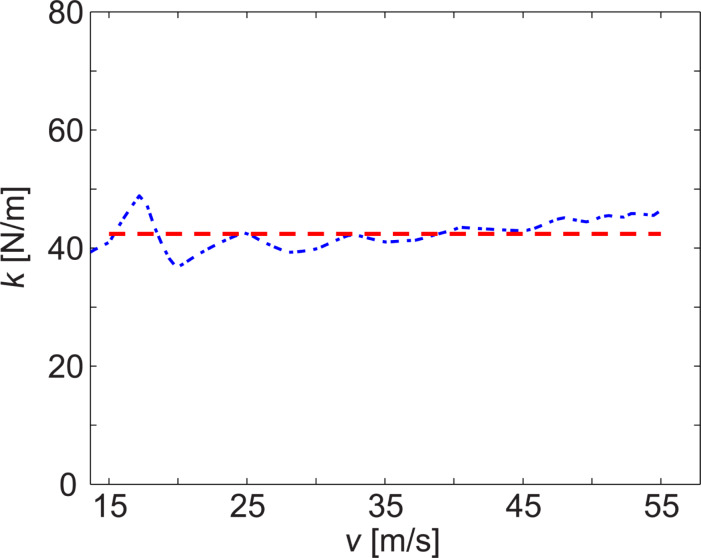
Experimentally determined 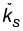
value for cantilever NCHV. The dashed line indicates the mean 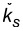 value for fluid speeds of 15–55 m/s.

To properly normalize the curve to zero deflection, the photodiode signal value at very low speeds was subtracted. Subsequently, Equation 3 was used to determine 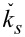. The result is shown in Figure 6. The highest deflection of NCHV was ≈52 nm in our experiments and therefore well within the linear response range of the cantilever [38].

This procedure was applied to all cantilevers studied. Table 2 summarizes the resulting spring constant values 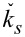 for fluid speeds above ≈15 m/s, together with their standard deviations.

For comparison, the spring constant values obtained by the thermal noise method (as described in [33]) are also provided, calculated according to

[6]
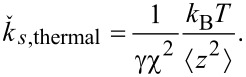


The dynamic-to-static optical lever sensitivity ratio, χ, was obtained with a self-coded MATLAB routine and was calculated for cantilevers with the geometrical dimensions reported in Table 1.

## Discussion

### Force profiles and cantilever deflection

The force profiles and the corresponding force per unit length exerted on the cantilevers by the fluid flow depend on the geometry of the beam and its alignment, (i.e., the position of the cantilever relative to the channel exit). In the case of a constant force distribution, the force profile would largely mirror variations in the cantilever width. As can be seen in Figure 3, the profiles show some deviation from this behavior. The length of some of the cantilevers (such as NCHV) is greater than the channel height and hence the force experienced by the cantilever decreases towards its fixed end. The picket-shaped end of cantilever NCHV, however, is clearly reflected in the force profiles displayed in Figure 3.

It is noteworthy that the position of the maximum force per unit length does not coincide with the center of the channel. This is also due to the chosen alignment in our experiment: the fluid escaping the channel must flow around the cantilever structure and the chip holding the cantilever. The fluid does not flow underneath the chip due to the small (≈100 μm) distance between the bottom of the chip and top edge of the microchannel and moves away from the vertical face of the cantilever chip. This moves the maximum force in the direction of the free end.

The presence of a tip on the cantilever was found to have negligible influence on the force profile and hence the total applied force: simulations of the forces applied to the cantilevers showed no significant difference in the total applied force when simulated with and without a tip. This should not be a surprise as the main fluidic force experienced by the cantilever is due to plan-view-dependent drag as opposed to viscous shear, and the former is not significantly influenced by the presence of the tip.

The reason for the observed hysteresis in the speed-dependent deflection (Figure 5) is not entirely clear. Some cantilevers showed no hysteresis at all while others showed more pronounced hysteresis, in particular Tap525. A change in the alignment of the cantilever relative to the channel had no effect on the observed phenomenon. The hysteresis could be related to changes in the humidity surrounding the cantilever as the fluid flow of dry nitrogen from the microchannel acts to decrease humidity with increasing fluid speed [8]. The metal coating on some of the cantilevers might also delaminate or let water enter, inducing some stress and causing additional bending, which is removed when nitrogen from the channel decreases the surrounding humidity. Checking the quality factors *Q* and the resonance frequencies of the resonance peaks for very low flow speeds at the beginning of the measurements and at the end did not reveal significant differences. If there is some stress induced, it is certainly small and not revealed by the *Q*-factor or the resonance frequency value.

### Conversion factors α, β and γ

The conversion factor γ, which relates the dynamic and static spring constants for a point load, is identical to one of the conversion factors required in relation to the thermal noise method. α and β are specific for our experiment and setup. α is a factor in relation to a dynamic-to-static optical lever sensitivity conversion. It is similar to the factor χ required for the thermal noise method [34]. The term “dynamic” in both cases refers to thermal oscillations and the first flexural mode. The term “static” refers to the optical lever sensitivity (in relation to the application of a point load at the free end in case of χ) and is related to the optical sensitivity linked to the force distribution resulting from the microchannel flow in case of α. χ and α are a measure of how much the cantilever bends at the free end compared to its bending in the first flexural mode for a given deflection at the free end. The conversion factor χ > 1 because an applied point load at the free end leads to a higher bending compared to the first flexural mode. In contrast, α < 1 because for the chosen cantilever alignment and the resulting force distribution, the bending is lower compared to the bending of the modal shape of the first flexural mode. Note that the α values are close to unity however (Table 2).

It has been reported in the literature that an analytical expression for β can be obtained for some force distributions and cantilever geometries [31]. For a constant force distribution and a rectangular beam β = 3/8 = 0.375. It can be seen that the values in Table 2 are of similar size because the cantilever shapes are similar to rectangular ones and the resulting force distributions due to the fluid flow show some similarity to a constant force distribution.

α and β depend on the force distribution and change if the force profile changes, for example, with fluid speed or cantilever positioning. However, Figure 4 and Table 2 demonstrate that there is no dramatic change in the values of both conversion factors (<3%) for any of the cantilevers studied and over the fluid speed ranges utilized to determine the spring constants. The highest changes are observed for the OTESPA and Fastscan-C cantilevers. The speed dependence of the conversion factors has been taken into account in the determination of the spring constant values reported in Table 2. Using the average values of α and β reported in Table 2 will give similar values to those reported for all cantilevers and fluid speeds above 15 m/s. Very small deviations would result for OTESPA and Fastscan-C cantilevers, where the values however should still give accurate results for fluid speeds around 25 m/s, corresponding to the speed where the mean values coincide with the calculated values.

### Spring constant values

Figure 6 shows a slight increase of the spring constant value 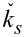 with fluid speed for cantilever NCHV. Ideally the curve should be a flat line. Not all cantilevers showed such an increase, which could be due to a small deviation from the modelled setup because of a slight misalignment of the cantilever or an angle between fluid flow and cantilever in the experiment that is slightly different from the one in the modelling. Another parameter where a small error would lead to this type of behavior is the normalization of the experimentally measured deflection curve to zero deflection. This is another reason, in addition to the observed hysteresis at lower fluid speeds for some of the cantilevers, why only speed values above 15 m/s were considered. The absolute error associated with the zero deflection normalization is the same for all deflections *d*_V_. As a result, the relative error in 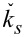 will be larger for small deflections, as described by Equation 5. Note, however, that maximum deflection values were in the range of ≈230 nm (Fastscan-C) to ≈17 nm (Tap525) and hence well within the linear response range of the cantilevers and the detector [38]. The size of the deflection itself should therefore not induce an error.

Some of the determined spring constant values show a significant deviation from the nominal values provided by the manufacturer. It is well known that such a discrepancy between the nominal and the actual values can exist [4,39]. All spring constants determined by the fluid flow method are however within the manufacturers quoted range, while for the thermal noise method, only the value for Tap150 falls slightly outside the nominal range.

The biggest deviation between the spring constants determined via thermal noise and the fluid flow method are observed for RC2 and Fastscan-C cantilevers. RC2 is a tipless cantilever and contact between the cantilever and a surface during the force curve required for the thermal noise method is in general not well defined. Therefore, there might be a large error in the experimental optical lever sensitivity, σ_1_, and hence in this particular thermal noise spring constant value. The optical lever sensitivity, when determined from force curves, has a potentially significant error even when contact between the cantilever and a surface is established with a tip [19]. In contrast, the method based on the fluidic force does not require this type of measurement.

Most of the spring constant values determined with the fluid flow are higher than those from thermal noise with the exception of RC2 and Tap150. A systematic error such as a misalignment of the cantilever relative to the flow could lead to such a deviation. A misalignment would result in a difference between the force experienced by the cantilever during the experiment in comparison to the force obtained from modelling, although it appears unlikely that such a potential systematic error was then not present for RC2 and Tap150.

An advantage of the presented approach, as compared to most other calibration methods, is that it gives the spring constant value for a range of applied forces and deflections with standard deviation values of typically <5%, while other methods often produce a value based on a single deflection.

The accessible spring constant range is not limited to the range 0.8–155 N/m of the present study. Softer cantilevers require a lower fluid flow speed and stiffer ones a higher speed. In order to have better control over the flow and the resulting forces, the channel height could be reduced for spring constant values <1 N/m and increased for cantilevers with spring constants >100 N/m.

### Microfluidic force tool

In the current experiments the specific alignment of the cantilevers relative to the channel exit was chosen for reasons of confidence in positioning and reproducibility. The positioning could however be further optimized for example by aligning the cantilever along the channel width (which was ≈1 mm in our experimental setup) such that the flow and hence force is essentially constant over the full cantilever length. This would potentially simplify the calculation of β. No simulation of the forces will be required if the total force depends on the plan view area and the fluid speed only, in which case forces can be predicted without any modeling. This is currently under investigation. We note that in the current setup, the *k* values for speeds above 15 m/s are already based on fluid flow profiles that essentially only depend on the speed of the fluid flow, which is evident from the very weak fluid speed dependence of α and β.

In addition to the determination of the static flexural spring constant, the presented setup can also be employed to obtain other useful information: knowledge of the static spring constant and the applied force allows the cantilever deflection to be determined, which in turn can be exploited to extract the torsional and lateral spring constants from the same measurement if the corresponding resonant frequencies are recorded [30]. Furthermore, the setup also allows the linear range of the force constants to be systematically tested for all kinds of cantilever sensors and other micro- and nanomechanical structures.

## Conclusion

We have demonstrated that a microfluidic gas flow escaping from a microchannel can be employed to provide accurate forces on the micrometer scale. We showed that a wide range of microcantilevers with very different static spring constants and geometric shapes can be calibrated without the need to bring the sensor into contact with a surface. An array of cantilevers could also easily be calibrated with the force tool described. The setup presents a contactless microfluidic force tool, which is generally applicable on small scales and has the potential to be equally useful in combination with smaller sensors and structures. The method should therefore be equally applicable to nanocantilever sensors and nanostructures.

## Supporting Information

File 1 Raw data of thermal noise spectra, cantilever deflection under fluid flow, and sensitivity values σ_1_.
