# Supplementary material for: Contact-free experimental determination of the static flexural spring constant of cantilever sensors using a microfluidic force tool
Source: Beilstein J Nanotechnol. 2016 Mar 30;7:492–500. doi: 10.3762/bjnano.7.43 (PMC4901535; doi:10.3762/bjnano.7.43)
Supplement: File 1 — Raw data of thermal noise spectra, cantilever deflection under fluid flow, and sensitivity values σ1. [file Beilstein_J_Nanotechnol-07-492-s001.zip › Supporting information and raw data/Supporting information.pdf]

Supporting information

Contact-free experimental determination of the static flexural spring constants of cantilever sensors using a microfluidic force tool

John D Parkin and Georg Hähner*

Address: *EaStCHEM* School of Chemistry, University of St. Andrews, North Haugh, St. Andrews, KY16 9ST, UK

Sensitivity factors, σ_1, were determined from force curves as described in the text:

	RA2	RC2	OTESPA	Tap150	NCHV	Tap525	Fastscan-C
Deflection sensitivity σ_1 (nm/V)	150.2	158.4	150.9	111.7	256.7	134.0	73.0

Raw data files:

File name: RA2um_perpg2_h100_ch120_offset0_tune1_2
File Format: TXT (ASCII)
Description: thermal resonance curve (power spectral density) for RA2

File name: RA2um-2-perpg2_pvsPD
File Format: LVM (ASCII)
Description: cantilever deflection in V vs pressure applied to microchannel in kPa for RA2

File name: RC2um_perpg2_h100_ch120_offset0_tune1_2
File Format: TXT (ASCII)
Description: thermal resonance curve (power spectral density) for RC2

File name: RC2um-1-perpg2_pvsPD
File Format: LVM (ASCII)
Description: cantilever deflection in V vs pressure applied to microchannel in kPa for RC2

File name: OTESPA-R3-0-perpg2-h100_ch100_offset0_thermal1_2
File Format: TXT (ASCII)
Description: thermal resonance curve (power spectral density) for OTESPA

File name: OTESPA-R3-1-perpg2-h100_ch100_offset0_pvsPD
File Format: LVM (ASCII)
Description: cantilever deflection in V vs pressure applied to microchannel in kPa for OTESPA

File name: Tap150A-0-Chip6_perpg2_h100_ch100_offset0_Tune1_1
File Format: TXT (ASCII)
Description: thermal resonance curve (power spectral density) for Tap150

File name: Tap150A-1-chip6_perpg2_h100_ch100_offset0_pvsPD
File Format: LVM (ASCII)
Description: cantilever deflection in V vs pressure applied to microchannel in kPa for Tap150

File name: NCHV-0-Chip3_perpg2_h100_ch100_offset0_Tune1_2_256_7nmV
File Format: TXT (ASCII)
Description: thermal resonance curve (power spectral density) for NCHV

File name: NCHV-1-chip3_perpg2_h100_ch100_offset0_pvsPD
File Format: LVM (ASCII)
Description: cantilever deflection in V vs pressure applied to microchannel in kPa for NCHV

File name: Tap525A-0-Chip2_perpg2_h100_ch100_offset0_Tune1_1
File Format: TXT (ASCII)
Description: thermal resonance curve (power spectral density) for Tap525

File name: Tap525A-2-chip2_perpg2_h100_ch100_offset0_pvsPD
File Format: LVM (ASCII)
Description: cantilever deflection in V vs pressure applied to microchannel in kPa for Tap525

File name: perpg2_h100_ch100_FastscanC_chip10_Tune1_1.txt
File Format: TXT (ASCII)
Description: thermal resonance curve (power spectral density) for FastscanC

File name: FastscanC-2-chip10_perpg2_h100_ch100_offset0_pvsPD.lvm
File Format: LVM (ASCII)
Description: cantilever deflection in V vs pressure applied to microchannel in kPa for FastscanC
